# The Treatment of Uric-Acid Gravel

**Published:** 1901-02-16

**Authors:** 


### THE TREATMENT OF URIC-ACID GRAVEL.

Dr. Gee, writing in the St. Bartholomew's Hospital *Journal*, says that he has found a very efficacious remedy for uric-acid gravel in the use of fresh whey as a drink. A large teacupful taken after each meal has not yet failed, he says, so far as his present experience goes, in preventing the appearance of uric-acid gravel, even in persons who have been subject to it for a considerable time. This seems a safe and simple remedy enough, and one well worthy of a trial.

